# Phosphatidylserine-targeted liposome for enhanced glioma-selective imaging

**DOI:** 10.18632/oncotarget.9584

**Published:** 2016-05-25

**Authors:** Liang Zhang, Amyn A. Habib, Dawen Zhao

**Affiliations:** ^1^ Department of Radiology, University of Texas Southwestern Medical Center, Dallas, TX, USA; ^2^ Department of Neurology and Neurotherapeutics, University of Texas Southwestern Medical Center and the North Texas VA Medical Center, Dallas, TX, USA; ^3^ Department of Biomedical Engineering, Wake Forest School of Medicine, Winston Salem, NC, USA; ^4^ Department of Cancer Biology, Wake Forest School of Medicine, Winston Salem, NC, USA

**Keywords:** phosphatidylserine (PS), blood brain barrier (BBB), glioblastoma multiform, magnetic resonance imaging (MRI), tumor vasculature

## Abstract

Phosphatidylserine (PS), which is normally intracellular, becomes exposed on the outer surface of viable endothelial cells (ECs) of tumor vasculature. Utilizing a PS-targeting antibody, we have recently established a PS-targeted liposomal (PS-L) nanoplatform that has demonstrated to be highly tumor-selective. Because of the vascular lumen-exposed PS that is immediately accessible without a need to penetrate the intact blood brain barrier (BBB), we hypothesize that the systemically administered PS-L binds specifically to tumor vascular ECs, becomes subsequently internalized into the cells and then enables its cargos to be efficiently delivered to glioma parenchyma. To test this, we exploited the dual MRI/optical imaging contrast agents-loaded PS-L and injected it intravenously into mice bearing intracranial U87 glioma. At 24 h, both *in vivo* optical imaging and MRI depicted enhanced tumor contrast, distinct from the surrounding normal brain. Intriguingly, longitudinal MRI revealed temporal and spatial intratumoral distribution of the PS-L by following MRI contrast changes, which appeared punctate in tumor periphery at an earlier time point (4 h), but became clustering and disseminated throughout the tumor at 24 h post injection. Importantly, glioma-targeting specificity of the PS-L was antigen specific, since a control probe of irrelevant specificity showed minimal accumulation in the glioma. Together, these results indicate that the PS-L nanoplatform enables the enhanced, glioma-targeted delivery of imaging contrast agents by crossing the tumor BBB efficiently, which may also serve as a useful nanoplatform for anti-glioma drugs.

## INTRODUCTION

Glioblastoma multiform (GBM) is the most common and lethal primary brain cancer. Median survival for GBM patients is 12 months even with the most aggressive treatment [1–3]. The standard of care for GBM is surgical resection followed by concurrent chemotherapy and radiation [2, 4]. Despite the slight improvement in GBM survival when adding chemotherapeutic temozolomide (TMZ), recurrences are inevitable. Many other potent chemotherapeutic agents that show high efficiency against extracranial tumors, however, are found to have limited efficacy on intracranial brain tumors, mainly due to the blood brain barrier (BBB) preventing these agents from reaching the tumor in brain parenchyma. Even though GBM tumors are composed of highly angiogenic and leaky microvessels, many GBM cells grow by co-opting the pre-existing vessels, where the BBB may remain intact [5, 6]. Thus, disruption of BBB in GBM is considered highly heterogeneous with many intratumoral regions still containing the intact BBB [6–9].

Much effort has been made to seek a solution for improved drug delivery to brain tumors. Nanoparticle system may represent an optimal carrier for brain delivery as its large payloads and the notable EPR (enhanced permeability and retention) effect has already been appreciated in clinical treatment of visceral cancers. However, as described above, the unique brain tumor vasculature that is distinct from that of extracranial tumors makes it impossible to deliver therapeutic doses of anti-cancer drugs to brain tumors simply based on nanoparticles via the EPR effect. Functionalization of nanocarriers with appropriate surface modification is a promising approach for the delivery of therapeutics across the BBB [9–11]. Taking advantage of receptors such as transferrin, insulin or low-density lipoprotein that are constitutively expressed on the BBB, ligands or antibodies specific to the receptors have been utilized to enable receptor-mediated transport of nano-drugs across the BBB [12]. Despite increased drug delivery to the tumor, there is a concern that such receptors are widespread, not tumor-specific, which may cause cytotoxicity to normal brain [13].

Clearly, discovery of a glioma-specific biomarker will be critical for development of a glioma-targeted nano-delivery system. Moreover, this biomarker needs to be accessible to its ligands or antibodies. Thus, a vascular luminal surface-exposed biomarker may be ideal. Previous studies, including by our group, have identified that phosphatidylserine (PS), the most abundant anionic phospholipid becomes exposed on the outer surface of vascular endothelial cells (ECs) of glioma due to oxidative stresses present in the tumor microenvironment [14–16]. Unlike tumor angiogenic markers such as α_v_β_3_ integrin, PS exposure is found in both the angiogenic and the existing non-angiogenic tumor vessels [17]. This is important, in particular for brain tumors because the non-angiogenic, pre-existing tumor vessels may remain intact and impermeable to therapeutics. The PS-exposed tumor vascular ECs are actually viable and not subject to apoptotic process. PS exposure on tumor cells is inducible and reversible; the cells can resume growth and reestablish phospholipid asymmetry, which is distinct from the irreversible process occurring in cell death [18, 19]. In normal mammalian cells, essentially all the PS localizes in the cell's inner membrane. Collectively, PS exposure on the luminal surface of tumor, but not normal blood vessels in the brain establishes a highly specific biomarker for glioma.

**Figure 1:**
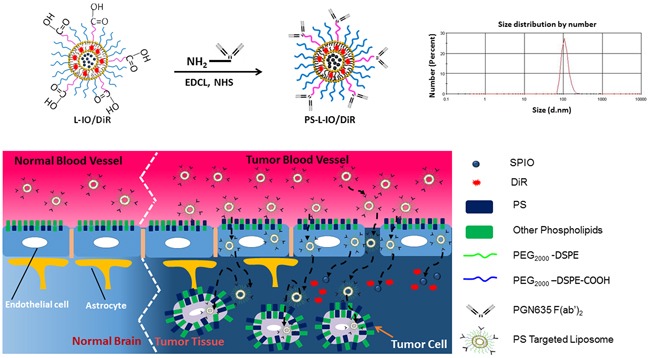
Illustrations of nanoformulation of PS-L-IO/DiR and its mode of action of the PS-targeted delivery for enhanced glioma-selective imaging A size distribution curve obtained by dynamic light scattering (DLS) analysis indicates an average hydrodynamic size of 110 nm.

We have recently established a PS-targeted nanoplatform (PS-L) by functionalizing the PEGylated liposomes with the F(ab')_2_ fragments of PGN635, a novel human monoclonal PS-targeting antibody. Our initial applications have confirmed its specific binding to PS-exposed tumor vascular ECs and tumor cells and subsequent internalization into the cells, enabling enhanced delivery of imaging contrast agents to extracranial tumors including U87 glioma implanted subcutaneously in mice [15, 20]. Encouraged by these preliminary studies, in the present study, we tested the capability of the PS-L to penetrate the BBB for glioma-targeted delivery in an orthotopic glioma mouse model. Our working hypothesis, as illustrated in Figure 1, is that PGN635 F(ab')_2_ leads the PS-L nanocarriers to bind to exposed PS on tumor vascular ECs and tumor cells that are accessible through the locally disrupted BBB in the intracranial glioma; for those ECs bound PS-L, subsequent endocytosis and incursion through the intact BBB results in glioma-selective delivery. As proof of concept, we loaded dual imaging contrast agents, iron oxide nanoparticles (SPIO) and near-infrared fluorescence dye, DiR into the PS-L (PS-L-IO/DiR), aiming to apply *in vivo* MRI and optical imaging for non-invasive assessment of its tumor-targeting specificity and longitudinal biodistribution.

## RESULTS

### Characteristics of PS-L-IO/DiR

The PS-targeting probe, PS-L-IO/DiR and its control probe, Aur-L-IO/DiR were characterized with respect to particle size, charge, encapsulation efficiency (EE) and antibody coupling efficiency (BCA assay and Bradford protein assay). The data are presented in Table 1 and Figure 1, which are highly consistent with our previous examinations of the same nanoprobes [20]. When the EE was calculated, the value was 57% and 98% for SPIO and DiR, respectively. Measurements of MRI relaxivity showed that the nanoprobe had a strong spin-spin relaxivity (r_2_ = 172 mM^−1^s^−1^) at 9.4T, and concentration-dependent T_2_ reduction was observed in the cells treated with PS-L-IO/DiR, but not Aur-L-IO/DiR (Supplementary Figure S1). Stability and toxicity studies were performed, showing that the liposomal nanoprobes were stable in serum for at least 48 h and minimally toxic to adult bovine aorta endothelial cells (data not shown).

**Table 1:** Characteristics of the bimodal liposomes functionalized with antibody F(ab')_2_

Nanoprobe	DiR loaded (μg)/ lipid (mg)	SPIO loaded (μg)/ lipid (mg)	Antibody modified (nmol)/ lipid (mg)	Mean size (nm)	Zeta potential (mV)	Polydispersity Index
PGN-L-IO/DiR	19.68±0.55	72.71±0.82	11.03±0.21	109.98±0.54	−3.89±0.10	0.11±0.01
Aur-L-IO/DiR	19.29±0.76	72.17±0.78	10.96±0.40	110.33±0.98	−3.74±0.21	0.11±0.01

Data were represented as the mean ± standard deviation. Each assay was repeated thrice.

### *In vitro* studies of PS-targeting specificity of PS-L-IO/DiR

**Figure 2:**
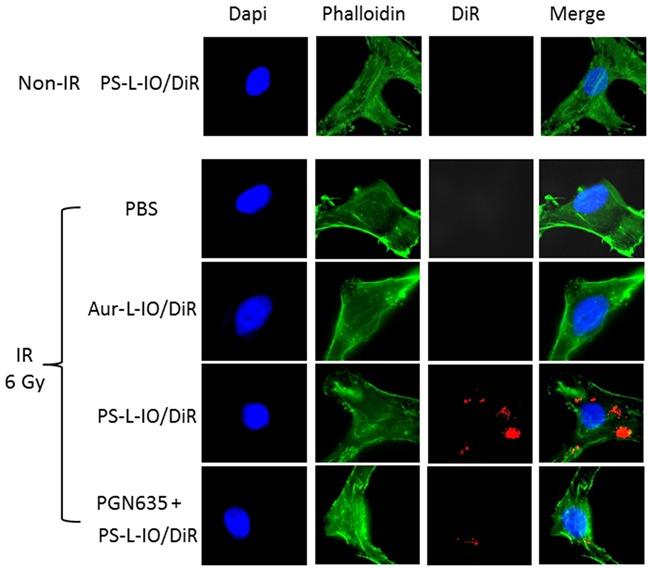
*In vitro* PS-targeting specificity of PS-L-IO/DiR Glioma U87MG-luc cells were pre-treated with/without a 6 Gy irradiation 24 h before they were incubated with PBS, Aur-L-IO/DiR or PS-L-IO/DiR for 1 h. Immunocytofluorescence images (40 X) of nuclei (Dapi) and cytoskeleton (green) staining were merged with DiR (red) signals of the same field, showing massive intracellular DiR signals only observed in the cells treated with PS-L-IO/DiR. Prior treatment with the full length PGN635 blocked majority of DiR signals.

Under normal culture conditions, there was essentially no PS exposed on the outer layer of U87-luc cell membrane (Figure 2). To induce PS exposure, the U87-luc cells were irradiated 24 h earlier with a single dose of 6 Gy. In good agreement with our previous studies [15, 20], robust fluorescence signals of DiR were detected in cytoplasm of the cells incubated with PS-L-IO/DiR, indicating that the initially cell membrane bound PS-L-IO/DiR became subsequently internalized (Figure 2). There was no intracellular DiR seen in the PBS or the control Aur-L-IO/DiR incubated cells (Figure 2). Specificity of PS-L-IO/DiR was further confirmed by pre-incubating the cells with non-labeled PGN635 to block the binding of PS-L-IO/DiR, which resulted in significant reduction in DiR signals of cell cytoplasm (Figure 2).

### Intracranial growth of orthotopic glioma and PS exposure on tumor vascular endothelial cells

**Figure 3:**
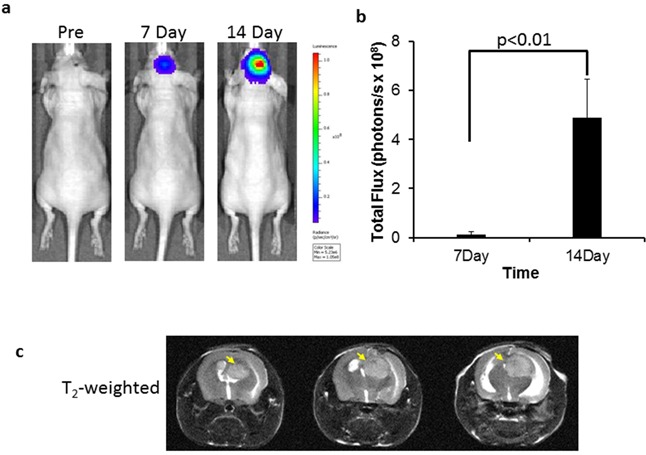
BLI and MRI monitoring of intracranial growth of U87 glioma **a.** Longitudinal BLI showed that light intensity in the mouse brain increased significantly over time, which was quantified in **b.** (p < 0.01). **c.** For the same mouse, T_2_-w MRI on day 14 revealed the hyperintense intracranial tumor on three consecutive slices (arrows). Hydrocephalus was also evident due to the tumor mass effect.

Seven days after intracranial implantation, an intracranial signal began to be detected by bioluminescence imaging (BLI), which became stronger on the follow-up on day 14 (Figure 3a). The mean light intensity of brain tumor detected on day 14 for the group of tumor-bearing mice was significantly greater than that on day 7 (4.90×10^8^ versus 1.43×10^7^ photons/s; p<0.01; Figure 3b). T_2_-weighted MR images confirmed the hyperintense intracranial tumor located in the right hemisphere of the mouse brain (Figure 3c), accompanied with significant hydrocephalus. *In vivo* quantification of PS exposure was conducted based on our previously described method [14]. Thirty-one percent (31 ± 6%) of tumor vascular ECs were found to have PS exposure.

### *In vivo* MRI and optical imaging of the PS-targeted bimodal nanoprobe

#### *In vivo* MRI

**Figure 4:**
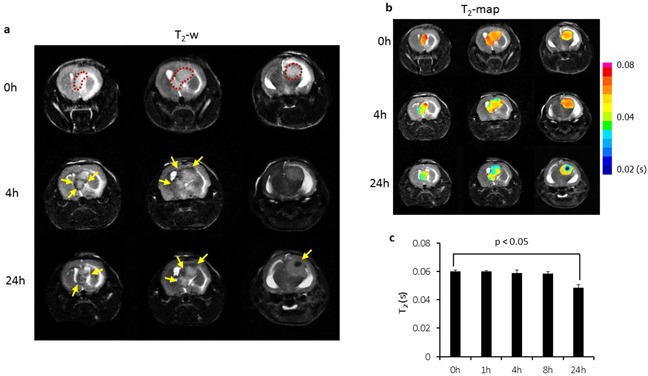
Longitudinal MRI of intratumoral biodistribution of the PS-targeted nanoprobe in orthotopic U87 glioma **a.** Representative T_2_-weighted fast spin echo multi-slice images were acquired before and at different time points post injection of PS-L-IO/DiR (2.5 mg Fe/kg) via a tail vein of a mouse bearing orthotopic U87 glioma. The hyperintense tumor was depicted (outlined) on 3 consecutive sections before injection. At 4 h post injection, signal voids (arrows) started to be seen primarily in tumor periphery (arrows), which became more apparent in both the peripheral and central tumor (arrows) at 24 h. **b.** T_2_ maps of the intracranial tumor at pre and post-injection were created and overlapped on T_2_-w images, indicating decreased T_2_ values over time. **c.** Quantitative T_2_ data for the group of tumors (n = 6) treated with PS-L-IO/DiR showed a significant shortening of mean T_2_ at 24 h (51 ± 2 (s.d.) ms), as compared to 61± 4 ms at the baseline (p<0.05).

**Figure 5:**
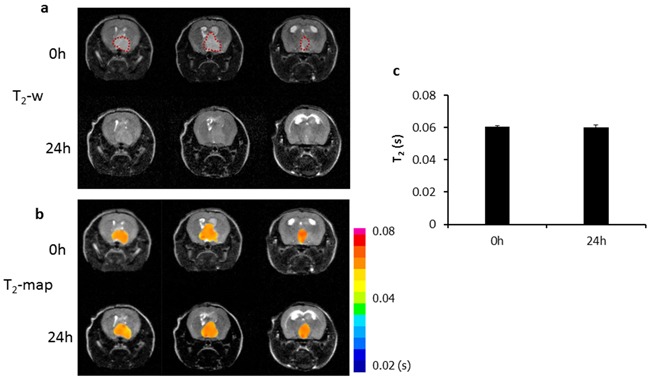
*In vivo* MRI of the control probe, Aur-L-IO/DiR **a.** To establish the glioma-targeting specificity of the PS-L-IO/DiR probe, the control probe Aur-L-IO/DiR (2.5 mg Fe/kg) was injected into a U87 glioma-bearing mouse. T_2_-weighted images of the mouse brain acquired before and 24 h post injection revealed no obvious signal change in the tumor region (outlined) at 24 h. T_2_ maps of the tumor **b.** and quantitative T_2_ data **c.** showed minimal change in T_2_ at 24 h (mean =60 ± 2) versus the baseline 0 h (61 ± 1 ms).

Longitudinal MRI was performed on the orthotopic U87 glioma bearing mice (n = 6) before and at different time points after i.v. injection of PS-L-IO/DiR (2.5 mg/kg iron). As shown in Figure 4a, a hyperintense intracranial tumor mass was depicted on T_2_-weighted images, accompanied with severe hydrocephalus. However, 4 h after PS-L-IO/DiR, punctate hypointense MRI signals started to appear within the tumor, which localized primarily in the tumor periphery. At 24 h, the intratumoral hypointense regions increased in number, intriguingly, many of the initially punctate regions were clustering to form larger regions of signal loss, which were seen in both the peripheral and central tumor. There was no detectable signal of PS-L-IO/DiR in the normal brain tissues (Figure 4a). T_2_ maps of the tumor were generated and overlapped on the anatomic T_2_-w images (Figure 4b). Quantitative T_2_ data of the group of tumors (n = 6) showed that T_2_ of the tumors shortened over time after injection of PS-L-IO/DiR (mean = 59 ± 3 ms at 4 h) and became significantly shorter at 24 h (mean = 51 ± 2 ms), as compared to the baseline at 0 h (mean = 61 ± 4 ms; p<0.05; Figure 4c). We further studied the *in vivo* glioma-targeting specificity of PS-L-IO/DiR by administering the control antibody conjugates, Aur-L-IO/DiR. As shown in Figure 5, there was a minimal change in MRI signals or T_2_ maps post Aur-L-IO/DiR (Figures 5a and 5b), which was confirmed with quantitative T_2_ of the Aur-L-IO/DiR group (n = 4; mean = 61 ± 1 ms at 0 h vs 60 ± 2 ms at 24 h; Figure 5c). From the results, it is apparent that the PS-targeted antibodies are necessary for this liposomal nanoplatform to penetrate the BBB/blood tumor barrier (BTB) and disseminate throughout the tumor parenchyma.

#### *In vivo* near infrared optical imaging

**Figure 6:**
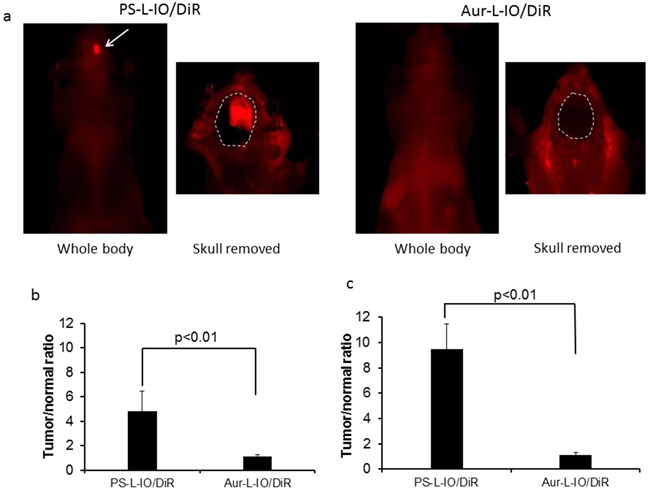
*In vivo* near infrared optical imaging of the PS-L-IO/DiR for specific glioma targeting Immediately after MRI at 24 h, *in vivo* optical imaging was conducted. **a.** For the same mouse shown in Figure 4, 24 h post PS-L-IO/DiR, the whole body NIR optical imaging revealed a brighter signal from the right brain. The region of brain tumor became clearly delineated from normal brain (outlined) after removal of the skull. There was no detectable tumor signal in the Aur-L-IO/DiR mouse brain even after the skull removal. **b.** Quantitative photon counts of the tumor and the contralateral normal brain were obtained from the whole body imaging. Tumor-versus-contralateral normal brain ratio (TNR) showed that mean TNR of the PS-L-IO/DiR group (mean = 4.8 ± 1.6; n = 6) was significantly higher than that of the Aur-L-IO/DiR treated mice (mean = 1.1 ± 0.1, p < 0.01). **c.** Removal of the skull significantly increased TNR (mean = 9.5 ± 1.9, p < 0.01) of the PS-L-IO/DiR group.

Immediately after MRI at 24 h, the mice were imaged with near infrared (NIR) optical imaging. As presented in Figure 6a, for the same mouse presented in Figure 4a, a clear optical signal emitting from the head of the mouse receiving PS-L-IO/DiR was captured in a whole body imaging setting. By contrast, there was no distinguishable contrast observed from the head of the mouse injected with Aur-L-IO/DiR (Figure 6a). To confirm that the signal was originating from the brain tumor, a surgical procedure was performed on the anesthetized mouse to remove the skull to expose the brain. A brighter and more focused light signal was emitted from the region of brain tumor in the PS-L-IO/DiR mouse (Figure 6a). No tumor contrast was seen in the brain of the Aur-L-IO/DiR mouse even after the skull removal (Figure 6a). Quantitative photon counts for the tumor and the contralateral normal brain were obtained and the tumor/normal ratio (TNR) was calculated. The mean TNR, obtained from the intact whole body imaging, was 4.8 ± 1.6 for the PS-L-IO/DiR group (n = 6), which was significantly higher than that of the control Aur-L-IO/DiR group (TNR=1.1 ± 0.1; n = 4; p<0.01; Figure 6b). Removal of the scalp and skull increased the TNR of the PS-L-IO/DiR group (mean TNR = 9.5 ± 1.9), but not that of the Aur-L-IO/DiR group (TNR=1.1 ± 0.2; p<0.01; Figure 6c). Concurring with the MRI data, the optical data further demonstrated the glioma-targeting specificity of PS-L-IO/DiR.

### *Ex vivo* optical imaging of biodistribution of the nanoprobes

**Figure 7:**
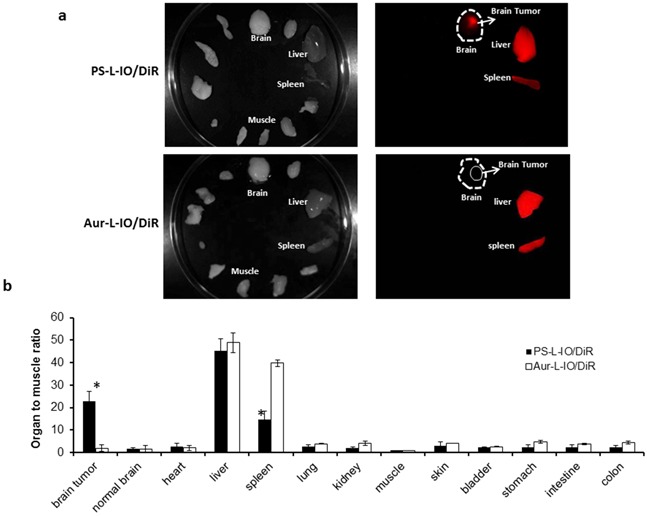
*Ex vivo* optical imaging of biodistribution of the PS-L-IO/DiR nanoprobe **a.** Immediately after *in vivo* optical imaging at 24 h, the glioma-bearing brain and various organs were dissected and subjected to *ex vivo* optical imaging. Under the NIR fluorescent light, the brain tumor was distinct from the normal brain in the mouse treated with the PS-L-IO/DiR, while it was not visible with the Aur-L-IO/DiR probe. Significant amounts of light signals were also captured from liver and spleen for each probe. **b.** Average photon counts for the tumor and each normal tissue were obtained and normalized by the muscle value (TMR), showing that TMR of brain tumors in the PS-L-IO/DiR treated mice was significantly higher than that of the Aur-L-IO/DiR mice (22.8 ± 4.5 vs 1.9 ± 1.2, *p < 0.01). The uptake of PS-L-IO/DiR by liver was slightly lower than that of the Aur-L-IO/DiR (45.3 ± 5.2 vs 48.9 ± 3.8); however, the spleen had significantly lower uptake of PS-L-IO/DiR (TMR = 14.6 ± 3.8 vs 39.8 ± 1.5, *p < 0.01).

*Ex vivo* NIR imaging was conducted on the excised tumor-bearing brain and normal tissues and organs immediately after *in vivo* imaging at 24 h (Figure 7a). The photon counts for the tumor and each organ were obtained and normalized to the muscle value (TMR). As shown in Figure 7b, mean TMR of the brain tumors in the mice injected with the PS-L-IO/DiR was as high as 22.8 ± 4.5, which was significantly higher than that of the Aur-L-IO/DiR (TMR = 2.0 ± 1.1, p < 0.01). As expected, significant amounts of both the PS-L-IO/DiR and the control Aur-L-IO/DiR nanoprobes were also taken up by liver and spleen of reticuloendothelial system (RES). However, compared to the Aur-L-IO/DiR, the glioma-targeted PS-L-IO/DiR showed significantly reduced accumulation in spleen (TMR = 14.6 ± 3.8 vs 39.8 ± 1.5, p < 0.01; Figure 7a and 7b).

### Histological and immunohistochemical validation

**Figure 8:**
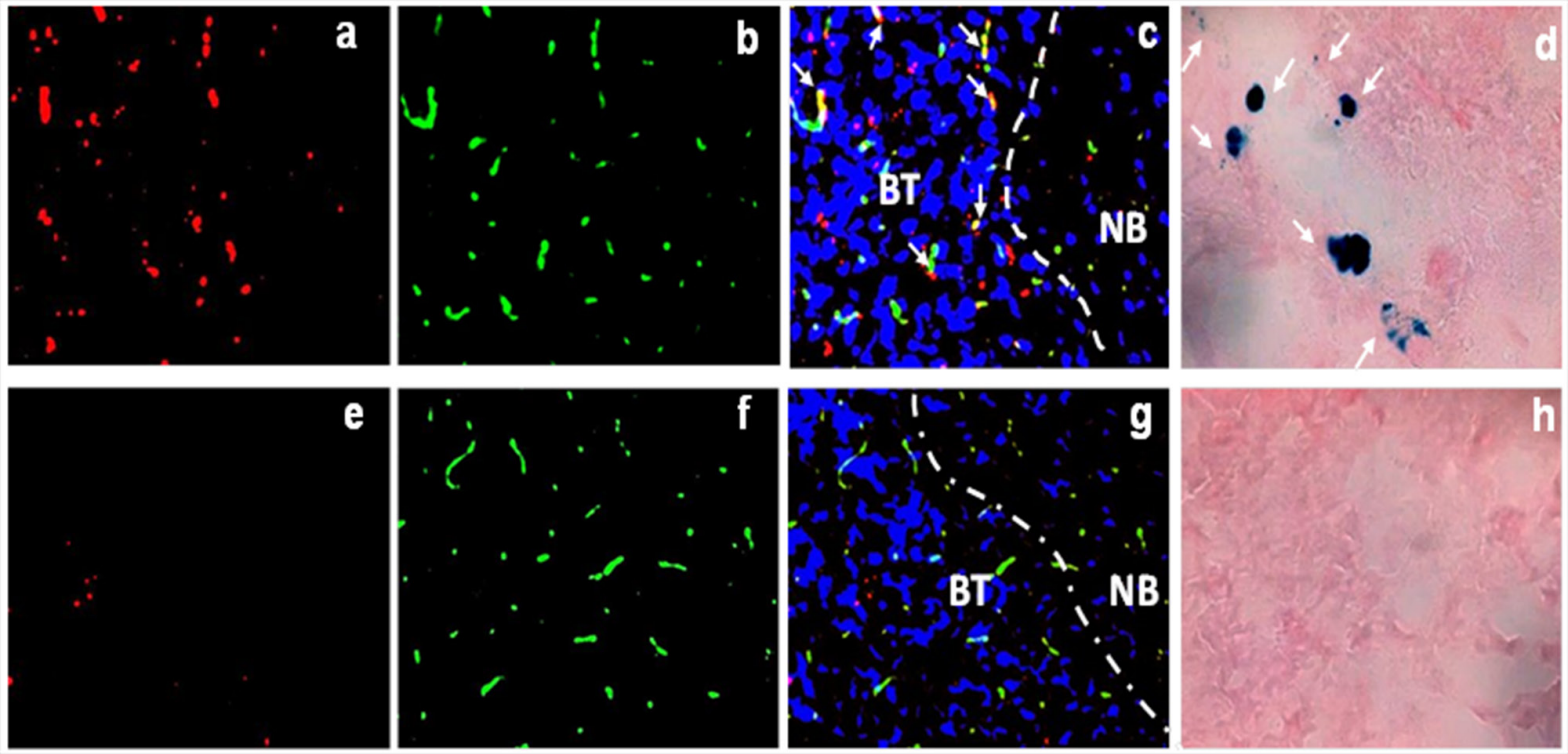
Fluorescence microscopy to detect the localization of PS-L-IO/DiR Frozen sections of tumor-bearing brain tissues were immunofluorescently examined for the localization of DiR signals (red) of the PS-L-IO/DiR **a-c.** and the control Aur-L-IO/DiR **e-g.** probe. Vascular endothelial cells were detected by immunofluorescence staining with anti-CD31 (green). Nuclei were detected with DAPI (blue). The merged image **c.** clearly showed that PS-L-IO/DiR localized predominantly in brain tumor tissues, with many co-localizing with tumor vascular endothelial cells (yellow, arrows). By contrast, there was much fewer, sparse DiR signals of the Aur-L-IO/DiR in tumor tissues **g.** Similarly, Prussian blue staining showed more blue-stained iron nanoparticles in the tumor treated with the PS-L-IO/DiR (arrows, **d.**) than the control Aur-L-IO/DiR **h.** Note: BT, brain tumor; NB: normal brain; Dotted line: boundary between tumor and normal brain. Magnification 20 X.

To validate the above *in vivo* imaging results, immunofluorescence and Prussian blue staining were conducted to detect DiR dye and iron oxide nanoparticles, respectively in the cryosections prepared from tumor-bearing brain tissues 24 h post i.v. injection of PS-L-IO/DiR or Aur-L-IO/DiR. As shown in Figure 8, abundant PS-L-IO/DiR nanoprobes were seen throughout the brain tumor (Figure 8a), whereas minimal DiR signal was found in the adjacent normal brain (Figure 8c). By contrast, few Aur-L-IO/DiR were seen in tumor tissues (Figures 8e and 8g). The data support the notion that at least small fractions of intratumoral nanoprobes result from the enhanced permeability and retention (EPR) effect via the locally disrupted BBB/BTB in tumor. Co-staining of vascular endothelial cells (ECs) with anti-CD31 antibody clearly revealed that many of the PS-L-IO/DiR nanoprobes co-localized with tumor vascular ECs, while many others infiltrated throughout extravascular tumor tissues (Figure 8c), further indicating the underlying mechanism for most of the PS-L-IO/DiR conjugates that bind specifically to the PS-exposed ECs and subsequently become internalized and eventually undergo transcytosis across the BBB. Similar results shown in the Prussian blue staining of iron nanoparticles (Figures 8d and 8h) are further supportive of the glioma-targeting specificity of the PS-L-IO/DiR nanoplatform.

## DISCUSSION

Currently, delivery of diagnostic/therapeutic agents to brain tumors remains a formidable challenge. Despite extensive angiogenesis in GBM, disruption of the BBB in GBM is heterogeneous, indicating that many intratumoral regions still contain intact BBB [6, 7]. Thus, nanocarriers with appropriate surface modification will be necessary in order to achieve the delivery of sufficient therapeutics to glioma in brain parenchyma via the BBB [10, 11, 21]. In the present study, we demonstrate that functionalization of the PEGylated liposome with a novel PS-targeting antibody, PGN635 F(ab')_2_ that binds specifically to tumor vascular ECs of glioma, enabled the dual imaging contrast agents-loaded PS-L (PS-L-IO/DiR) to be delivered selectively and efficiently to tumor tissues, but not the normal brain. This was clearly visualized by both the *in vivo* optical imaging and MRI, and validated by *ex vivo* optical imaging and fluorescence microscopy. Moreover, minimal accumulation of the control antibody labeled nanoplatform, Aur-L- IO/DiR in glioma concurs with previous observations that permeability of non-targeting nanoparticles through the BBB in glioma is limited, further supporting that the mechanism of action of the PS-L to penetrate the tumor BBB is via its specific binding to exposed PS on tumor vasculature.

In addition to sensitive detection of specific tumor targeting, MRI provided spatial information about intratumoral localizations of PS-target nanoprobes. In contrast to the T_1_ contrast agent, gadolinium-DTPA, SPIO encapsulated in the core of liposome is a T_2_ contrast agent, which generates negative contrast on T_2_ or T_2_^*^-weighted images. Compared to T_1_ contrast agents, SPIO has much higher molar relaxivity, thus, is widely used for molecular imaging applications by MRI [22–24].

Unlike the relatively homogeneous enhancement seen by optical imaging (Figures 6 and 7), MRI revealed heterogeneous T_2_-w signal loss and T_2_ reduction in the intracranial tumor (Figure 4). Importantly, longitudinal MRI data, as shown in Figure 4, also postulated the pharmacokinetics and pharmacodistribution of the PS-L-IO/DiR in the intracranial gliomas. At an earlier time 4 h after systemic administration, the punctate signal voids caused by the susceptibility effect of SPIO were seen primarily at tumor periphery on T_2_-w MRI. The noticeable pattern of punctate MRI signal changes may result from the vascular phase of PS-L-IO/DiR when significant numbers of the circulating PS-L-IO/DiR are bound to the PS exposed on tumor vascular ECs but have not yet penetrated the vessels. The timing of the vascular phase actually coincided well with our previous studies of using PGN635 to localize to exposed PS in tumors, in which our histopathological analysis determined massive PGN635 binding to tumor vascular ECs at 4 h [14, 16]. Following the vascular phase, the tissue phase was occurring over time after subsequent internalization by ECs, and followed by extravasation through the tumor BBB and extravascular tissue distribution of the PS-L-IO/DiR, which was reflected as the clustering dark signals that were widespread in both tumor center and periphery on T_2_-w images MRI at 24 h (Figure 4). The extravascular localization of massive DiR signals observed by fluorescent microscopy and the clustering iron nanoparticles evidenced by Prussian blue staining was strongly supportive for the tissue phase of the PS-L-IO/DiR (Figure 8). Furthermore, lack of marked signal changes in the glioma treated with the control Aur-L-IO/DiR negated the possibility that intratumoral accumulation of PS-L-IO/DiR was simply due to the EPR effect via the leaky glioma vasculature (Figure 5). This observation is in a good agreement with the study by Sarin et al. suggesting that the upper limit of the pore size of blood-brain tumor barrier in a rodent glioma model is ~12 nm [25]. The larger particles such as ~100 nm used in our study, if used without targeting moieties, are too big to pass through the small pores of the tumor BBB.

Once the BBB is traversed, cargos of nanoparticles need to be disseminated across the glioma parenchyma to exert their therapeutic effects on individual tumor cells. Diffusibility of these nanoencapsulated agents in brain/tumor parenchyma constitutes a further challenge for drug delivery to the central nervous system (CNS). It has been suggested in a rat brain study that the nanoparticles need to be small enough to penetrate efficiently within the brain parenchyma through the pores (pore size < 64 nm) of the extravascular extracellular space [26]. However, Nance, et al. have recently shown that with appropriate PEG coating and a neutral surface charge, the actual size of nanoparticles can go up to 114 nm without significantly affecting their movement within brain tissues [27]. In our present study, the PEGylated PS-L-IO/DiR with a mean size of 110 nm and near a neutral surface charge (Table 1) falls well into the range of the aforementioned physical criteria, which may also contribute to the wide distribution of the PS-L-IO/DiR in glioma.

Most clinically approved nanocarrier drugs are liposomal formulations. A number of chemical and physical properties of the liposome nanocarrier are suitable for drug delivery to tumor tissues. As already discussed above, the size of liposome is highly related to its tumor permeability and intratumoral diffusibility. Having taken into account a combination of important factors including loading capacity, clearance, tumor vascular permeability and tissue diffusion, it has been generally accepted that an optimal liposome size for effective drug delivery should be at ~ 100nm [28]. Indeed, both Doxil and LEP-ETU have the size in the range of 100-150nm. Nevertheless, after systemic administration, only a fraction of the dose localizes at the target site, while most of the injected doses end up in the RES of liver and spleen. As expected, our *ex vivo* biodistribution study showed that the non-targeted Aur-L-IO/DiR were found mostly in liver and spleen (Figure 7). Even with the PS-targeted delivery, the liver still had the highest uptake of the PS-L-IO/DiR despite significant increase in tumor uptake. Intriguingly, the spleen uptake of PS-L-IO/DiR decreased significantly, as compared to Aur-L-IO/DiR (Figure 7). Similar findings have been observed in our previous studies in a breast cancer mouse model [20].

The novel, fully human monoclonal PS-targeting antibody, PGN635, recognizes PS in a β2-glycoprotein I (β_2_GP1)-dependent fashion. PGN635 binds to PS complexed with the PS-binding protein, β2GP1 with a higher specificity and affinity to PS (Kd ≈ 10^−10^ M), as compared to annexin V [14, 29, 30]. PGN635 has a more restricted specificity for PS than does annexin V, which recognizes PE in addition to PS and other anionic phospholipids. Moreover, annexin V has a different binding site from the PS-targeting antibody, thus does not compete with the PS-targeting antibody [17]. We have previously shown that 27% of the vessels of U87 gliomas have exposed PS, while increased levels of PS exposure were found in the gliomas treated with chemotherapeutics or irradiation (65%) [14, 31]. PS exposure is also known for its role in prothrombin anchoring and activation of the coagulation cascade by platelets [32], which may contribute to the formation of multi-focal necrosis, one of the characteristics of clinical GBM. As PS exposure is exclusive to tumor vascular ECs, it can thus serve as a useful biomarker for GBM. In addition to PS-targeting antibodies, alternative PS-binding agents have been explored. Blanco et al. showed that the nanoformulated saposin C (SapC) and dioleoylphosphatidylserine (DOPS) triggered the death of glioma cells after SapC bound to PS exposed glioma cells and tumor vascular ECs [33, 34]. Aiming to achieve image-guided drug delivery to glioma, we have successfully integrated the anti-cancer chemotherapeutic agents into the MRI contrast agent loaded PS-L nanoplatform. Our preliminary studies have shown that the delivery and release of the nano-drugs to glioma cells can be monitored in real time by MRI, and efficient delivery of the nano-drugs lead to massive deaths of TMZ-resistant glioma cells [31].

## MATERIALS AND METHODS

### Preparation and characterization of PS-targeted liposomes loaded with MRI and optical imaging contrast

The procedure used for preparation of liposomes has been described previously [20]. In brief, egg phosphatidylcholine (EPC) (Sigma-Aldrich Corporation, MO), cholesterol (Sigma-Aldrich Corporation, MO), 1,2-distearoyl-sn-glycero-3-phosphoethanolamine-N-[methoxy(polyethylene glycol)2000] (PEG2000–DSPE), 1,2-distearoyl-sn-glycero-3-phosphoethanolamine-N-[carboxy(polyethylene glycol)2000] (COOH-PEG2000-DSPE) (Avanti Polar Lipids, Alabaster, AL) and 1,1′-dioctadecyl-3,3,3′,3′-tetramethyl indotricarbocyanine Iodide (DiR, Perkin-Elmer, Waltham, MA) were dissolved in chloroform (53:45:4:1:0.8 μmol/μmol) in a pear-shaped flask. The lipid film was prepared by removing the chloroform using a rotary vacuum evaporator. The rough DiR plus SPIO liposomes were produced by hydration of the film with PBS containing SPIO (10 nm, Ocean Nanotech, AR) with sonication in water bath for 5 min, followed by sonication using a probe-type sonicator (Omni International Inc., Kennesaw, GA) for 5 min. The sample was centrifuged at 1000 g for 15 min at 4°C to precipitate the free SPIO. Unencapsulated DiR in the supernatant was further removed by using a DynaMag™ (Invitrogen, Grand Island, NY) magnetic separator overnight at 4°C. The DiR plus SPIO liposomes were suspended and extruded through polycarbonate membranes with 400 nm and then 200 nm pore sizes (Nalgene, Rochester, NY). The content of SPIO in the liposomes was determined by the atomic absorption spectrometer (Varian Medical Systems, USA), and the content of DiR in the liposomes was determined by the microplate reader (Molecular Devices, Sunnyvale, CA). The encapsulation efficiency (EE) of SPIO or DiR was determined based on the formula: EE = (W_encap_ / W_total_) × 100%, where W_encap_ is the measured amount of SPIO or DiR in the liposomal suspensions, W_total_ is the total amount of SPIO or DiR used initially.

To functionalize the bimodal liposomes with PS-targeting antibody, the human monoclonal antibody PGN635 that was generated by Affitech A.S. (Oslo, Norway) in collaboration with Peregrine Pharmaceuticals. Inc. (Tustin, CA) was used. Aurexis is a human monoclonal antibody that binds to an irrelevant antigen (Staphylococcus aureus clumping factor A) and was used as a negative control antibody. PGN635 and Aurexis F(ab')_2_ fragments were generated by reacting antibodies with pepsin at a molar ratio of 1:130 (antibody:pepsin) for 1 h at 37°C. F(ab')_2_ fragments (MW = 110 kD) were purified by FPLC using an S-200 column (Pharmacia, Piscataway, NJ) and PBS running buffer. PGN635 F(ab')_2_ or aurexis F(ab')_2_ were then conjugated to the distant terminus of polyethylene glycol (PEG) chain coated liposomes (Figure 1). Briefly, 3.52 μmol of 1-(3-dimethylaminopropyl)-3-ethylcarbodiimide hydrochloride (EDC, Sigma-Aldrich) and 7.88 μmol of N-hydroxysuccinimide (NHS, Sigma Aldrich) were added into 2 ml of DiR and SPIO loaded liposomes (COOH-PEG2000-DSPE:EDCI:NHS = 0.02:3.52:7.88, μmol/μmol; pH 7.4) and gently stirred for 30 min at room temperature. PGN635 F(ab')_2_ or aurexis F(ab')_2_ (PGN635 F(ab')_2_ or aurexis F(ab')_2_: COOH-PEG2000-DSPE = 1:2, μmol/μmol) were then added into the suspensions and allowed to continuously react for 5 h at room temperature. Excess EDC and NHS were removed by dialysis. The liposome suspensions were applied to a quick spin Sephadex G-50 column (Fisher Scientific, Pittsburg, PA) equilibrated with PBS and centrifuged at 150 g at 4°C to remove the uncoupled antibodies. Subsequently, liposome fractions were collected from quick spin Sephadex G-50 column, and the content of antibodies on the liposomes was determined by both bicinchoninic acid (BCA) assay and Bradford protein assay (Sigma-Aldrich). The nanoprobes are referred to PS-L-IO/DiR or the control, Aur-L-IO/DiR throughout the text.

The mean diameter and zeta potential of the liposome nanoprobes were measured by dynamic light scattering (DLS) analysis with Zetasizer 3000HSA (Malvern Instruments Ltd., U.K.). The stability of the nanoprobes was evaluated using a simple filtration test. In brief, PS-L-IO/DiR or Aur-L-IO/DiR in PBS (pH 7.4) containing 10% fetal bovine serum was incubated for 48 h at 37°C. 100 ml of each solution was continuously passed through syringe filter membranes (Nalgene, USA) with different pore sizes of 0.80, 0.45, and 0.22 μm at 0 h, 24 h and 48 h, respectively. Near infrared fluorescence (NIRF) images of each sample before/after the filtration test were observed using a Maestro imaging system (CRi, Inc., Woburn, MA).

### Immunocytochemistry and MRI for *in vitro* binding specificity

Human glioma U87MG cells (ATCC, Manassas, VA, USA) were maintained in Dulbecco modified Eagle medium (DMEM) supplemented with 10% FBS. As described previously, U87MG cells were infected with a lentivirus containing a firefly luciferase reporter, and highly expressing stable U87-luc clones were isolated [35]. To induce PS exposure, the cells were treated with a single dose of 6 Gy X-radiation [20]. Twenty-four hours later, the cells with/without irradiation were incubated with PS-L-IO/DiR or the control Aur-L-IO/DiR at a concentration of 29 μg/ml iron with addition of β2 glycoprotein 1 (β2GP1) at the same concentration for 1 hr. For the blocking study, the cells were pretreated with the full length PGN635 for 1 h prior to PS-L-IO/DiR. Unbound particles were washed away with PBS, and then the cells were fixed with 4% paraformaldehyde (PFA). DiR signals were captured under fluorescence microscope and merged with the immunocytochemical staining of cytoskeleton with phalloidin (Alexa Fluor 488® phalloidin 1:500; Life Technology, Eugene, Oregon) and nuclei with Dapi. For MRI relaxivity study, the PS-L-IO/DiR or Aur-L-IO/DiR treated cells (3 × 10^5^) were mixed homogeneously with 0.8% agarose and fast spin echo multi-slice (FSEMS) T_2_-weighted images (TR = 3000 ms, effective TE ranging from 40 to 120 ms with a 20 ms increment, average = 2, number of slices = 10, acquisition time = 10 min) were acquired using a 9.4T horizontal bore magnet. Both T_2_-weighted SI and T_2_ values of each agarose mixture were quantified.

### Orthotopic glioma xenografts

All animal procedures were approved by the Institutional Animal Care and Use Committee of University of Texas Southwestern Medical Center and Wake Forest School of Medicine. A ~1 cm long incision of skin was made along the midline of the brain of an anesthetized nude mouse (BALB/c nu/nu; NCI, Frederick, MD). Using a high-speed drill, a 1 mm burr hole of the skull was made in the right hemisphere, anterior to the coronal fissure. About 5×10^4^ U87-luc cells in 4 μl mixture of PBS and Matrigel (25%, BD Biosciences, San Jose, CA) were injected directly into right caudal diencephalon, 1.5 mm beneath the dura mater using a 32G Hamilton syringe. The burr hole was filled with bone wax and the scalp was closed with sutures.

### *In vivo* BLI/ MRI for tumor detection

BLI was initiated for monitoring intracranial tumor growth 7 days after tumor implantation using the IVIS Spectrum system (Caliper, Xenogen, Alameda, CA). The tumor bearing mice (n = 6) were anesthetized (isoflurane/O_2_ in an induction chamber; isoflurane from Baxter International Inc., Deerfield, IL) and a solution of D-luciferin (120 mg/kg in PBS in a total volume of 80 ml; Biosynthesis, Naperville, IL) was administered s.c. in the neck region. Anesthesia was maintained with isoflurane (2%) in oxygen. Five minutes after luciferin injection, an array of various exposure times was applied for image acquisition. Data were quantified with the Living Imaging software by using absolute photon counts (photons/s) in an ROI, manually drawn to outline the BLI signal of the brain. Once a BLI signal was observed, MRI was performed to assess tumor volume and follow up its growth by using a 9.4 T horizontal bore magnet with a Varian INOVA Unity system (Palo Alto, CA). Each mouse was maintained under general anesthesia (2% isoflurane) during the scan. Fast spin echo multi-slice (FSEMS) sequence was applied (TR = 2500 ms, TE =100 ms, slice thickness = 1 mm, number of slice = 10, matrix = 256 × 256, field of view = 20 × 20 mm and average = 4) to acquire T_2_-weighted images.

### *In vivo* MRI and optical imaging of tumor targeted PS-L-IO/DiR nanoprobes

To study *in vivo* specific glioma targeting of PS-L-IO/DiR nanoprobes, longitudinal T_2_-weighted FSEMS MRI at 9.4 T was acquired before and at 1, 4, 8 and 24 h after i.v. injection of PS-L-IO/DiR (2.5 mg Fe/kg; n = 6) or the control Aur-L-IO/DiR (2.5 mg Fe/kg; n =4). T_2_-weighted FSEMS images (TR = 3000 ms, effective TE ranging from 40 to 120 ms with a 20 ms increment, average = 2, number of slices = 10, acquisition time = 10 min). Immediately after MRI at 24h, near infrared fluorescence imaging was performed using Maestro imaging system (CRI Inc. Woburn, MA). Immediately after the last image at 24 hours, the skull of the mouse was surgically removed to expose both hemispheres of the brain and a last *in vivo* image was obtained. The whole surgical procedure and imaging were completed within 10 minutes under anesthesia, and no obvious bleeding occurred. For MRI data, T_2_ measurements of tumors were quantified for each time point post i.v. injection of the liposomal nanoprobes. For optical imaging analysis, average photon counts normalized by time (s) in brain tumors and contralateral normal brain were obtained. TNR (tumor to normal ratio) was used for quantifying dynamic changes in signal intensity.

### *Ex vivo* optical imaging of biodistribution

Immediately after *in vivo* imaging, the tumor-bearing mice were sacrificed. Brain and major organs were dissected and subjected to optical imaging. Average photon counts were obtained for brain tumor, normal brain and each organ/tissue. The photon counts were further normalized by the muscle value.

### Histological and immunohistochemical studies

For fluorescence microscopy, cryosections of brain tissues bearing glioma were immunostained with antibodies against the endothelial marker, CD31 (BD Biosciences, San Jose, CA) and followed by Cy2-conjugated secondary antibody. The near infrared signals of DiR were recorded and merged with the CD31 image and the DAPI-stained image of the same field. Prussian blue staining was performed to stain iron and counterstained by nuclear fast red [20].

### Statistical analysis

Analysis of MRI data was performed on a home written MATLAB program on both pixel-by-pixel and region of interest (ROI) basis. ROIs were drawn on brain tumors on the high resolution T_2_-weighted images. Signal intensity (SI) in these ROIs was measured for all the echoes of FSEMS images. One-way ANOVA analysis of variance was used to determine significance among groups, after which post hoc tests with the Bonferroni correction were used for multiple comparisons between individual groups.

## CONCLUSIONS

Given the fact that many GBM blood vessels remain impermeable, the discovery and development of a drug delivery system that enables penetration specifically across the tumor BBB will be critical for effective glioma-targeted diagnosis and treatment. In the present study, we demonstrate that the PS-targeted liposomal (PS-L) nanoplatform allows sufficient delivery of the dual imaging contrast agents across the tumor BBB to the glioma parenchyma, which was visualized by *in vivo* MRI and optical imaging. The temporal and spatial changes in intratumoral MRI contrast may correlate well with the initial binding of the PS-L to tumor vascular ECs and disseminating across the tumor tissues at the later stage. The study thus provides proof of principle for the further development of glioma-targeted nanodelivery of therapeutic agents or theranostic agents for both glioma imaging and treatment.

## SUPPLEMENTARY FIGURE



## References

[1] Howlader N, Noone AM, Krapcho M, Garshell J, Neyman N, Altekruse SF, Kosary CL, Yu M, Ruhl J, Tatalovich Z, Cho H, Mariotto A, Lewis DR, Chen HS, Feuer EJ, CK (2013). SEER Cancer Statistics Review, 1975-2010.

[2] Wen PY, Kesari S (2008). Malignant gliomas in adults. N Engl J Med.

[3] Park S, Hatanpaa KJ, Xie Y, Mickey BE, Madden CJ, Raisanen JM, Ramnarain DB, Xiao G, Saha D, Boothman DA, Zhao D, Bachoo RM, Pieper RO, Habib AA (2009). The receptor interacting protein 1 inhibits p53 induction through NF-kappaB activation and confers a worse prognosis in glioblastoma. Cancer Res.

[4] Stupp R, Mason WP, van den Bent MJ, Weller M, Fisher B, Taphoorn MJ, Belanger K, Brandes AA, Marosi C, Bogdahn U, Curschmann J, Janzer RC, Ludwin SK (2005). Radiotherapy plus concomitant and adjuvant temozolomide for glioblastoma. N Engl J Med.

[5] di Tomaso E, Snuderl M, Kamoun WS, Duda DG, Auluck PK, Fazlollahi L, Andronesi OC, Frosch MP, Wen PY, Plotkin SR, Hedley-Whyte ET, Sorensen AG, Batchelor TT, Jain RK (2011). Glioblastoma recurrence after cediranib therapy in patients: lack of “rebound” revascularization as mode of escape. Cancer Res.

[6] Jain RK, di Tomaso E, Duda DG, Loeffler JS, Sorensen AG, Batchelor TT (2007). Angiogenesis in brain tumours. Nat Rev Neurosci.

[7] Kroll RA, Neuwelt EA (1998). Outwitting the blood-brain barrier for therapeutic purposes: osmotic opening and other means. Neurosurgery.

[8] Gilbertson RJ, Rich JN (2007). Making a tumour's bed: glioblastoma stem cells and the vascular niche. Nat Rev Cancer.

[9] Cheng Y, Dai Q, Morshed RA, Fan X, Wegscheid ML, Wainwright DA, Han Y, Zhang L, Auffinger B, Tobias AL, Rincon E, Thaci B, Ahmed AU, Warnke PC, He C, Lesniak MS (2014). Blood-brain barrier permeable gold nanoparticles: an efficient delivery platform for enhanced malignant glioma therapy and imaging. Small.

[10] Meyers JD, Doane T, Burda C, Basilion JP (2013). Nanoparticles for imaging and treating brain cancer. Nanomedicine (Lond).

[11] Zhang L, Zhao D (2014). Applications of nanoparticles for brain cancer imaging and therapy. J Biomed Nanotechnol.

[12] Kang T, Jiang M, Jiang D, Feng X, Yao J, Song Q, Chen H, Gao X, Chen J (2015). Enhancing Glioblastoma-Specific Penetration by Functionalization of Nanoparticles with an Iron-Mimic Peptide Targeting Transferrin/Transferrin Receptor Complex. Mol Pharm.

[13] Gabathuler R Approaches to transport therapeutic drugs across the blood-brain barrier to treat brain diseases. Neurobiol Dis.

[14] Zhao D, Stafford JH, Zhou H, Thorpe PE (2011). Near-infrared Optical Imaging of Exposed Phosphatidylserine in a Mouse Glioma Model. Transl Oncol.

[15] Zhang L, Zhao D (2013). Liposomal encapsulation enhances *in vivo* near infrared imaging of exposed phosphatidylserine in a mouse glioma model. Molecules.

[16] He J, Yin Y, Luster TA, Watkins L, Thorpe PE (2009). Antiphosphatidylserine antibody combined with irradiation damages tumor blood vessels and induces tumor immunity in a rat model of glioblastoma. Clin Cancer Res.

[17] Ran S, Downes A, Thorpe PE (2002). Increased exposure of anionic phospholipids on the surface of tumor blood vessels. Cancer Res.

[18] Ran S, Thorpe PE (2002). Phosphatidylserine is a marker of tumor vasculature and a potential target for cancer imaging and therapy. Int J Radiat Oncol Biol Phys.

[19] Mirnikjoo B, Balasubramanian K, Schroit AJ (2009). Mobilization of lysosomal calcium regulates the externalization of phosphatidylserine during apoptosis. J Biol Chem.

[20] Zhang L, Zhou H, Belzile O, Thorpe P, Zhao D (2014). Phosphatidylserine-targeted bimodal liposomal nanoparticles for *in vivo* imaging of breast cancer in mice. J Control Release.

[21] Gao X, Li C (2014). Nanoprobes visualizing gliomas by crossing the blood brain tumor barrier. Small.

[22] Bulte JW, Kraitchman DL (2004). Iron oxide MR contrast agents for molecular and cellular imaging. NMR Biomed.

[23] Thorek DL, Chen AK, Czupryna J, Tsourkas A (2006). Superparamagnetic iron oxide nanoparticle probes for molecular imaging. Ann Biomed Eng.

[24] Zhou H, Stafford JH, Hallac RR, Zhang L, Huang G, Mason RP, Gao J, Thorpe PE, Zhao D (2014). Phosphatidylserine-targeted molecular imaging of tumor vasculature by magnetic resonance imaging. J Biomed Nanotechnol.

[25] Sarin H, Kanevsky AS, Wu H, Brimacombe KR, Fung SH, Sousa AA, Auh S, Wilson CM, Sharma K, Aronova MA, Leapman RD, Griffiths GL, Hall MD (2008). Effective transvascular delivery of nanoparticles across the blood-brain tumor barrier into malignant glioma cells. J Transl Med.

[26] Thorne RG, Nicholson C (2006). *In vivo* diffusion analysis with quantum dots and dextrans predicts the width of brain extracellular space. Proc Natl Acad Sci U S A.

[27] Nance EA, Woodworth GF, Sailor KA, Shih TY, Xu Q, Swaminathan G, Xiang D, Eberhart C, Hanes J (2012). A dense poly(ethylene glycol) coating improves penetration of large polymeric nanoparticles within brain tissue. Sci Transl Med.

[28] Torchilin VP (2005). Recent advances with liposomes as pharmaceutical carriers. Nat Rev Drug Discov.

[29] Ran S, He J, Huang X, Soares M, Scothorn D, Thorpe PE (2005). Antitumor effects of a monoclonal antibody that binds anionic phospholipids on the surface of tumor blood vessels in mice. Clin Cancer Res.

[30] Luster TA, He J, Huang X, Maiti SN, Schroit AJ, de Groot PG, Thorpe PE (2006). Plasma protein beta-2-glycoprotein 1 mediates interaction between the anti-tumor monoclonal antibody 3G4 and anionic phospholipids on endothelial cells. J Biol Chem.

[31] Zhang L, Zhang Z, Mason RP, Sarkaria JN, Zhao D (2015). Convertible MRI contrast: Sensing the delivery and release of anti-glioma nano-drugs. Sci Rep.

[32] Versteeg HH, Heemskerk JW, Levi M, Reitsma PH (2013). New fundamentals in hemostasis. Physiol Rev.

[33] Blanco VM, Latif T, Chu Z, Qi X (2015). Imaging and Therapy of Pancreatic Cancer with Phosphatidylserine-Targeted Nanovesicles. Transl Oncol.

[34] Blanco VM, Chu Z, Vallabhapurapu SD, Sulaiman MK, Kendler A, Rixe O, Warnick RE, Franco RS, Qi X (2014). Phosphatidylserine-selective targeting and anticancer effects of SapC-DOPS nanovesicles on brain tumors. Oncotarget.

[35] Zhou H, Luby-Phelps K, Mickey BE, Habib AA, Mason RP, Zhao D (2009). Dynamic near-infrared optical imaging of 2-deoxyglucose uptake by intracranial glioma of athymic mice. PLoS One.

