# Supplementary material for: Phosphatidylserine-targeted liposome for enhanced glioma-selective imaging
Source: Oncotarget. 2016 May 25;7(25):38693–706. doi: 10.18632/oncotarget.9584 (PMC5122421; doi:10.18632/oncotarget.9584)
Supplement: Supplementary file 1 [file oncotarget-07-38693-s001.pdf]

Phosphatidylserine-targeted liposome for enhanced glioma-selective imaging

SUPPLEMENTARY FIGURE

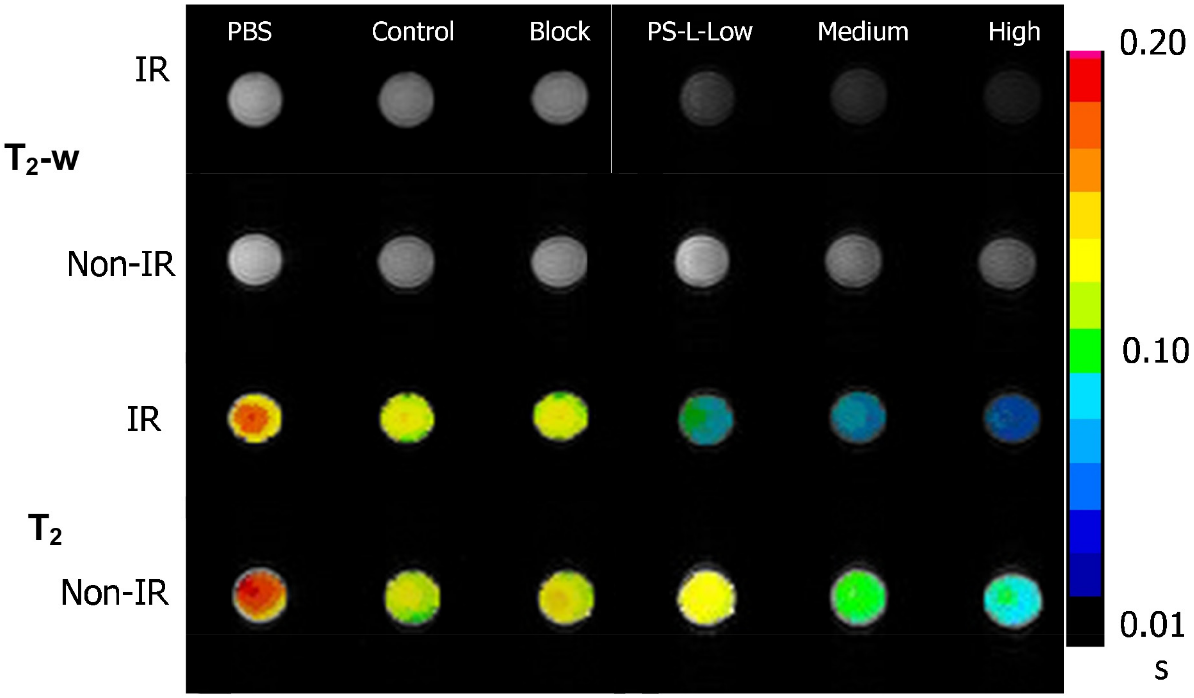

Supplementary Figure S1: Strong MRI T_2 effect of PS-L-IO/DiR. Top: T_2-weighted images clearly showed that the irradiated cells incubated with PS-L-IO/DiR appeared darker (decreased SI) and maximal reduction of SI was observed at the highest iron concentration (High; 52 µg/ml). There was no significant signal change in Aur-L-IO/DiR treated cells (Control) or non-irradiated cells. Specific binding of PS-L-IO/DiR to exposed PS was blocked by pre-treating the cells with unlabeled PGN635 antibodies (Block). Bottom: Corresponding maps of T_2 relaxation time showed significant reduction of T_2 values for the irradiated cells incubated with PS-L-IO/DiR.
